# Screening of the *LAMB2, WT1, NPHS1*, and *NPHS2* Genes in Pediatric Nephrotic Syndrome

**DOI:** 10.3389/fgene.2018.00214

**Published:** 2018-06-22

**Authors:** Aiysha Abid, Saba Shahid, Madiha Shakoor, Ali A. Lanewala, Seema Hashmi, Shagufta Khaliq

**Affiliations:** ^1^Centre for Human Genetics and Molecular Medicine, Sindh Institute of Urology and Transplantation, Karachi, Pakistan; ^2^Department of Genomic, National Institute of Blood Diseases, Karachi, Pakistan; ^3^Department of Human Genetics, University of Health Sciences, Lahore, Pakistan; ^4^Department of Pediatric Nephrology, Sindh Institute of Urology and Transplantation, Karachi, Pakistan

**Keywords:** nephrotic syndrome, *NPHS1*, *NPHS2*, *LAMB2*, *WT1*

## Abstract

Mutations in the *NPHS1, NPHS2, LAMB2*, and the *WT1* genes are responsible for causing nephrotic syndrome (NS) in two third of the early onset cases. This study was carried out to assess the frequencies of mutations in these genes in a cohort of pediatric NS patients. A total of 64 pediatric familial or sporadic SRNS cases were recruited. Among these, 74% had a disease onset of up to 3 years of age. We found one homozygous frameshift mutation in the *NPHS1* gene in one CNS case and two homozygous mutations in the *NPHS2* gene. Six mutations in four cases in the *LAMB2* gene were also identified. No mutation was detected in the *WT1* gene in isolated SRNS cases. *LAMB2* gene missense mutations were segregating in NS cases with no extra-renal abnormalities. Analysis of the population genomic data (1000 genome and gnomAD databases) for the prevalence estimation revealed that NS is more prevalent than previously determined from clinical cohorts especially in Asian population compared with overall world populations (prevalence worldwide was 1in 189036 and in South-Asian was 1in 56689). Our results reiterated a low prevalence of mutations in the *NPHS1, NPHS2, LAMB2*, and *WT1* genes in the studied population from Pakistan as compared to some European population that showed a high prevalence of mutations in these genes. This is a comprehensive screening of the genes causing early onset NS in sporadic and familial NS cases suggesting a more systematic and robust approach for mutation identification in all the 45 disease-causing genes in NS in our population is required.

## Introduction

Nephrotic syndrome (NS) is a common kidney disorder in children characterized by proteinuria, hematuria, hyperalbuminemia, and hypercholesterolemia. Pediatric NS is a heterogeneous disease that manifests early in life and termed as congenital (CNS), infantile, and childhood-onset NS according to the age of onset. A majority shows favorable response to the steroid treatment while about 20–30% of the children establish steroid resistant NS (SRNS) and rapidly progress to renal failure. Patients with inherited NS show poor renal survival but have a low disease-recurrence rate after kidney transplantation (Conlon et al., 1999).

Immunosuppression therapy has shown to be effective in 8–10% of the genetic cases (Bierzynska et al., 2015). Mutations in around 45 genes have been associated with familial and sporadic NS (Bierzynska et al., 2015). Several approaches are established for preferential screening of the genes based on the age of onset, inheritance patterns and histopathology. In a large study with isolated sporadic NS occurring within the first year of life, two third of the cases were due to the mutations in the *NPHS1, NPHS2, WT1*, and *LAMB2* genes (Hinkes et al., 2007). Mutations in the *NPHS1* and *NPHS2* genes together share a large proportion of mutations that cause NS in children. The other two genes, *WT1* and *LAMB2* have also been associated with the syndromic or complex forms (Löwik et al., 2009; Zenker et al., 2009).

NS is a major glomerular disease in Pakistani children accounted for 50–74% of all the cases presented with glomerular diseases in major kidney centers in the country (Ali et al., 2011; Imtiaz et al., 2016). Among these 30% of the cases develop steroid resistance (Mubarak et al., 2009) while 10–20% of the NS cases show steroid resistance according to published studies (Ruf et al., 2004; Weber et al., 2004). In a previous study, we observed low frequencies of mutations in the *NPHS1* and *NPHS2* genes in pediatric NS (Abid et al., 2012). Therefore, it was essential to perform a systematic screening of other genes causing NS in children. In view of the observations, presented by Hinkes et al. (2007), the current study was designed to ascertain the prevalence of mutations in the *LAMB2, WT1, NPHS1*, and the *NPHS2* genes to further explore the spectrum of disease-genes in Pakistani children with early onset NS.

Furthermore, we used publicly available whole genome/exome data to calculate population based estimates of the prevalence of NS worldwide and in our population. This study provides insight into the NS phenotypes and the prevalence and significance of particular alleles especially that are prevalent in our population.

## Materials and methods

### Patient recruitment and data collection

A total of 64 NS patients (ranging from congenital and childhood-onset) were selected from a cohort recruited from the pediatric nephrology department of the Sindh Institute of Urology and Transplantation Karachi. Among them 14 patients were of early onset NS (congenital to 3 years of age). The research protocol was approved by the Institutional Review Board and conformed with the tenets of the Declaration of Helsinki. Written informed consent was obtained from the parents of all the subjects. Patients with CNS, infantile and childhood onset NS including familial and sporadic cases that are younger than 16 years of age, resistant to standard steroid therapy were selected in this study.

NS was diagnosed by the presence of edema, urinary protein excretion equal to or greater than 40 mg/m^2^/h and serum albumin below 2.5 g/l. Renal failure was designated when estimated glomerular filtration rate (eGFR) was <90 ml/min by the Schwartz formula (Schwartz and Work, 2009). All the patients received standard steroid therapy on initial presentation. The clinical response to steroid therapy was classified as described as: (1) steroid sensitive; i.e., complete remission of proteinuria during the steroid therapy persisting for at least 12 weeks after therapy; (2) steroid dependent; i.e., remission of proteinuria during therapy but recurrence when the dosage was reduced below a critical level or relapse of proteinuria within the first 3 months after the end of therapy and (3) resistant; i.e., no remission of proteinuria during 4 consecutive weeks of daily steroid therapy.

### Mutation analysis

Blood samples were collected in ACD vacutainer tubes. Genomic DNA was extracted using the standard phenol-chloroform extraction procedure. Mutation analysis was performed by direct DNA sequencing of 29 exons of the *NPHS1* gene, 8 exons of the *NPHS2* gene, 11 exons of the *WT1* gene and 33 exons of the *LAMB2* gene. Genomic sequences of the genes were obtained from the Ensembl genome browser (Ensembl ID's: ENSG00000161270 for the *NPHS1*, ENSG00000116218 for the *NPHS2*, ENSG00000184937 for the *WT1* and ENSG00000172037 for the *LAMB2* genes respectively) and exon-specific intronic primers were designed in the forward and reverse directions and were obtained commercially (Eurofins MWG Operon, Germany). Each exon was individually amplified by PCR in a 25 μl reaction volume using 1 μg of genomic DNA under standard PCR conditions. Amplified PCR products were purified using the PCR clean-up kit (Promega Wizard®, Promega Corporation, Madison, WI, USA). The sequencing reaction was performed using the BigDye terminator cycle sequencing kit, V3.1 (Applied Biosystems®, California, USA). Sequencing products were purified using the Centri-Sep spin columns (Princeton Separation®) and were analyzed on an automated DNA analyzer (ABI, 3100). Each mutation was confirmed by repeat sequencing in both the forward and reverse orientations.

### Bioinformatics analysis

Each identified variant was assessed for the clinical interpretation of pathogenicity by using InterVar (http://wintervar.wglab.org; Li and Wang, 2017) and ClinVar (https://www.ncbi.nlm.nih.gov/clinvar; Landrum et al., 2016). To assess the damaging effects of missense mutations not previously reported in the Human Gene Mutation Database (HGMD public; http://www.hgmd.cf.ac.uk accessed 30-09-2016), *in silico* bioinformatic tools like PolyPhen-2 (v2; http://genetics.bwh.harvard.edu/pph2/index.shtml), SIFT (http://sift.jcvi.org/), and Condel (http://bg.upf.edu/fannsdb/; González-Pérez and López-Bigas, 2011) were employed. Splice-site mutations were predicted by NetGene 2 (http://www.cbs.dtu.dk/services/NetGene2/) server. A multiple sequence alignment (MSA) across different species was constructed using the CLUSTAL OMEGA tool (http://www.ebi.ac.uk/Tools/msa/clustalo/) which utilized BLOSUM algorithm for this purpose (Goujon et al., 2010). MSA was then used to calculate the evolutionary conservation scores of amino acid positions where mutations were found (Landau et al., 2005; Ashkenazy and Kliger, 2010) and also to construct the phylogenetic tree with MEGA 5 program (Tamura et al., 2011).

In an attempt to investigate the potential impact of mutations, an *in-silico* prediction on wild type and mutant protein was performed using the online protein modeling I-TASSER server (http://zhanglab.ccmb.med.umich.edu/I-TASSER) and were further analyzed for comparison for respective mutations using PyMOL Molecular Graphics System (Version 1.7.4.4 2010 Schrodinger, LLC; http://www.pymol.org). In the wild type protein model, the identified missense mutations were made by using the Discovery Studio Version 3.1. The amino acid change is visualized in the rotamer form with side chain orientations incorporated from Dunbrack backbone dependent rotamer library with maximum probabilities. For c.2673dupCA mutation in *NPHS1* gene, translation analysis was done by using Expasy translate tool.

The data for the estimation of prevalence and carrier frequencies (CF) were downloaded from the 1000 genome browser (http://1000genomes.org/ accessed 05-10-2017; The 1000 Genome Project Consortium et al., 2015) by using the Allele Frequency Calculator tool (http://grch37.ensembl.org/Multi/Tools/AlleleFrequency accessed 05-10-2017) and genome Aggregation database (gnomAD http://gnomad.broadinstitute.org/ accessed 07-10-2017; Lek et al., 2016) in VCF format. Known mutations within the *NPHS1, NPHS2*, and the *LAMB2* genes that were listed in the HGMD/ClinVar were annotated. All other non-synonymous/indel variants in the coding regions with MAF <0.01 were selected and were scored for potential pathogenicity using Condel tool. Using the reference allele count and alternate allele count, prevalence (1/q^2^) and carrier frequency (1/2pq) were calculated according to the Hardy-Weinberg Equilibrium for each gene variants by using the sum of all alternate *NPHS1, NPHS2*, and *LAMB2* alleles (also for known plus unknown scored as pathogenic according to Condel). Calculations for combined three genes frequencies were done by adding the p^2^ values of each gene's frequencies.

## Results

A total of 64 patients including early-onset (from congenital to 3 years of age) and childhood-onset NS (disease onset after 3 years of age) were screened for mutations in the *NPHS1, NPHS2, LAMB2*, and *WT1*genes. In the first part of the study, 14 early-onset cases were screened for mutation in the *NPHS1* and the *NPHS2* genes only. Three patients aged 0–3 years in this group were identified to have homozygous mutations in the *NPHS1* and the *NPHS2* genes. In the second part of the study, a total of 61 patients were included. Among these, 11 patients were from first part and 50 patients were selected from a previous cohort that was negative for the *NPHS1* and the *NPHS2* gene mutations (Abid et al., 2012). All these (*n* = 61) were then screened for mutations in the *LAMB2* and the *WT1* genes.

Clinical characteristics of the patients are given in Table 1. Clinical data were obtained for all the cases. Early-onset cases include congenital and infantile onset cases as well as children with disease onset of 2–3 years of age. Family history was positive in 20% of the cases, whereas, 80% cases were sporadic NS. Histopathological findings were available in majority of the cases that predominantly showed FSGS and MCD. Renal failure was established in 8 patients, of these three expired.

**Table 1:** Clinical characteristics of children with Nephrotic Syndrome.

Total number of children	64
Age of onset	Since birth−16 years
Females (%)	25 (39.0%)
Males (%)	39 (60.9%)
Male to Female ratio	1.52:1
**CLASSIFICATION OF NS**	
Early-onset NS (CNS/infantile/2–3years) (%)	47 (73.5%)
Childhood NS (%)	17 (26.5%)
**RENAL BIOPSY FINDINGS**	
FSGS^a^	16
MCD^b^	16
IgMN^c^	4
MesPGN^d^	4
MGN^e^	2
**FAMILY HISTORY**	
Positive (%)	13 (20.3%)
Negative (%)	51 (79.7%)
**OUTCOME**	
ESRD^f^/CRF^g^	5 (9.43%)
Lost to follow up	21 (18.8%)
Expired	3 (5.66%)

a Focal segmental glomerular sclerosis

b minimal change disease

c IgM nephropathy

d mesengial proliferative glomerulonephritis

e membranous glomerulonephritis

f end stage renal disease

g *chronic renal failure*.

### Mutation screening

**Figure 1:**
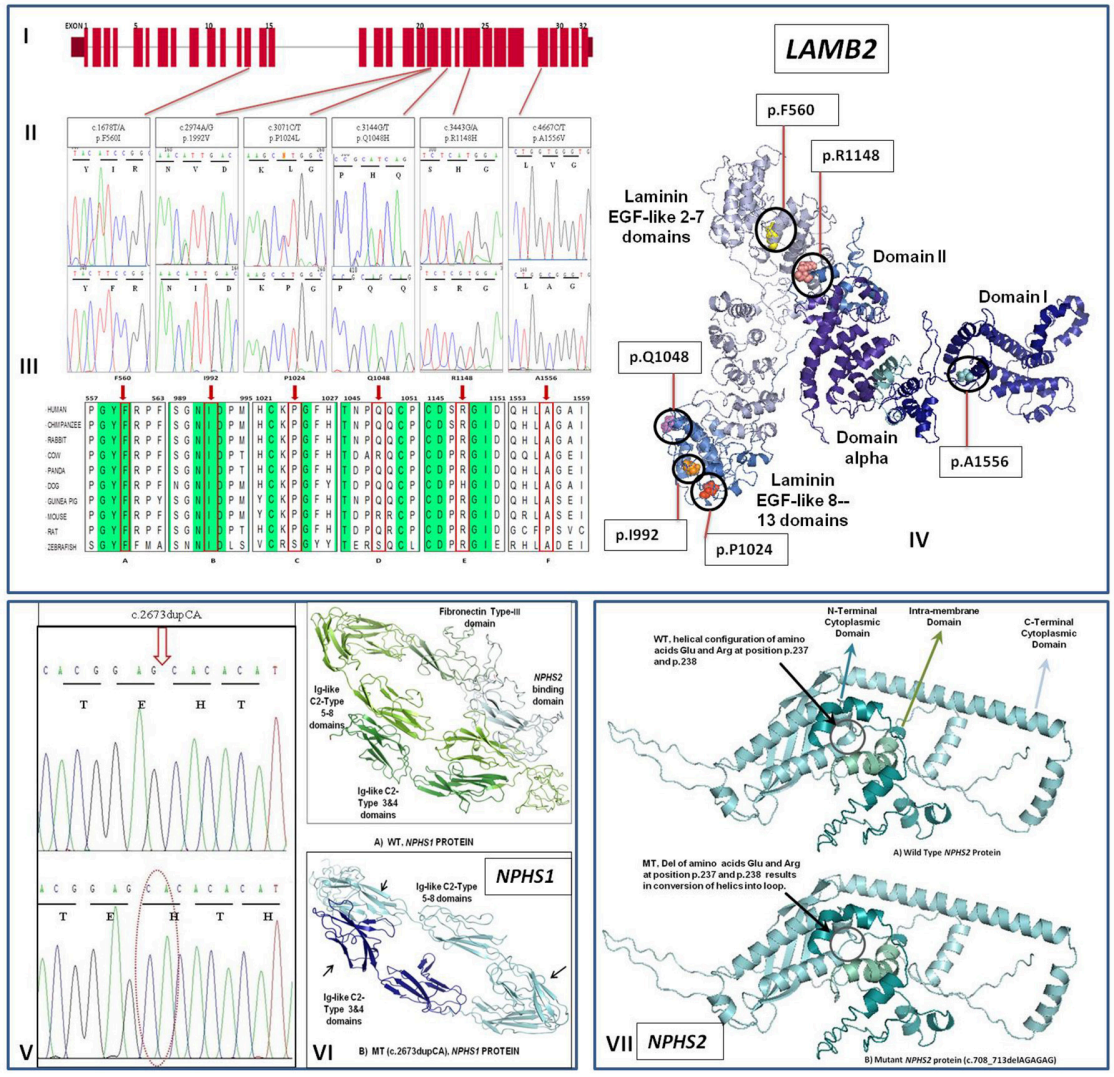
**(I)** Exon structure of human *LAMB2* gene contains 32 exons. **(II)** Sequence electropherogram showing mutated (upper panel) and normal (lower panel) sequences. **(III)** Phylogenetic tree and MSA for *LAMB2* gene showing sequence alignment of particular amino acid and its conservation in other orthologs. Conserved sequences are highlighted. (a) and (b) shows the highly conserved amino acids indicated by red frame and arrow at position 560 and 992, respectively while (c–f) shows that amino acids at position 1,024, 1,048, 1,148, and 1,556 are mostly conserved in other species. **(IV)** An illustration of Molecular distribution of *LAMB2* missense mutations in relation to the functional domains of the protein**. (V)** Representative electropherogram (lower panel) of mutation (c.2673dupCA) in the *NPHS1* gene compared with wild type sequence (upper panel). **(VI)** Conformational changes in the overall configuration of the mutant nephrin protein marked with arrows. **(VII)** Conformational changes in the overall configration of the mutant podocin protein (mutant positions are encircled). WT wild type, MT mutant protein.

We identified one homozygous mutation in the *NPHS1* gene in one CNS case, two homozygous mutations in the *NPHS2* gene in two early onset cases. Homozygous and compound heterozygous mutations were detected in four childhood onset cases in the *LAMB2* gene (Table 2, Figure 1). No mutation was discovered the *WT1* (entire coding sequence) gene screening.

**Table 2:** List of homozygous/compound heterozygous mutations identified.

**Patient**	**Sex**	**Family history**	**Age Onset (yrs)**	**Biopsy**	**cDNA**	**Protein**	**Response to therapy**	**Renal outcome/Time follow up**	**dbSNP/HGMD ID/ClinVar *In silico* prediction**
**LAMB2**									
NS001	M	No	10	IgMN^a^	c.1678T/A c.3071C/T	p.F560I p.P1024L	SRNS^b^	Expired with ESRD^c^	Not reported/not reported/pathogenic rs368506627/not reported/uncertain significance
NS032	F	yes	12	^d^FSGS	c.2974A/G c.3443G/A	p.I992V p.R1148H	SRNS	partial remission with CyA^e^	rs529614319/not reported/pathogenic rs138774635/not reported/benign
NS050	M	No	13.5	FSGS	c.3144G/T	p.Q1048H	SRNS	No response	Not reported/not reported/likely pathogenic
NS113	M	No	1	FSGS	c.4667C/T	p.A1556V	SRNS	Lost to follow up	rs774045808/CM066905/pathogenic
**NPHS1**									
NS301	M	yes	CNS	–	c.2673dupCA		SRNS	No response	Not reported/not reported/pathogenic
**NPHS2**									
NS313	M	No	2	FSGS	c.795-2A/G Splice-site	–	SRNS	Lost to follow up	Not reported/not reported/pathogenic splice-site aborted
NS304	M	No	3	MesPGN	c.708_713delAGAGAG	–	SRNS	ESRD	Not reported/not reported/pathogenic
**NPHS2 RISK ALLELE**									
NS228	M	No	2	MesPGN^f^	c.755G/A	p.R229Q	SRNS	No response	rs61747728/CM023107/likely pathogenic and risk factor
NS245	F	No	CNS	FSGS	c.755G/A	p.R229Q	SRNS	Partial remission	rs61747728/CM023107/likely pathogenic and risk factor

a IgM nephropathy

b Steroid resistant nephrotic syndrome

c end stage renal disease

d focal segmental glomerular sclerosis

e Cyclosporine A

f *mesengial proliferative glomerulonephritis*.

Analysis of the *NPHS1* gene revealed only one mutation (c.2673dupCA) in one congenital case that inherited one mutant copy of the gene from each parent (Table 2, Figure 1V). Translation analysis for homozygous c.2673dupCA mutation demonstrated that CA duplication at position 2673 in exon 20 results in shifting of the reading frame from position 893 with the introduction of premature stop codon at position 904 leads to protein truncation. Comparision of crystal diagram with wild type protein model (Figure 1VI) suggested the loss of both fibronectin type III and *NPHS2* binding domains in the mutated protein which include some extracellular region, transmembrane, and cytosolic region. These domains are responsible for the interaction with surrounding molecules to maintain the integrity of Slit diaphgram. Possibly loss of two these interacting domains may result in over all disruption of filtration barrier leading to proteinuria and steriod resistant nephrotic syndrome. A novel splice-site mutation (c.795-2A/G) which abolishes the splice-site in the *NPHS2* gene was identified in one child aged 2 years of age. The patient did not respond to standard steroid therapy and lost to follow up after 2 years of treatment. A homozygous deletion (c.708_713delAGAGAG) in the *NPHS2* gene was present in another early-onset NS patient. Deletion of two amino acids (R237, E238) as a result of this mutation changed the helical configuration into loop structure in the *NPHS2* protein structure (Figure 1VII). Two early onset cases also had p.R229Q mutation which is attributed as a risk factor for NS.

We did not find any mutation in the *WT1* gene in isolated early onset NS cases. The *LAMB2* gene screening revealed six homozygous or compound heterozygous mutations in four infantile and late-onset sporadic NS cases (Table 2, Figures 1I–IV). All *LAMB2* gene mutations were missense mutations. Among these three mutations were novel mutations (Table 2). All mutations were conserved in nature and were predicted to be pathogenic by *In-Silico* analysis (Figures 1I–IV). Only one mutation was identified in an infantile case. None of the CNS case showed mutation in the *LAMB2* gene. Pathogenicity, clinical correlation of mutations and responses to therapy of respective patients are given in Table 2. Ten infantile and early onset NS cases also exhibited single variants in the *NPHS1* and the *LAMB2* genes (Table 3). None of the above mutations were found in 50 healthy controls. Minor allele frequencies (MAF) of these variants when reported was > 0.01 with no homozygous detected in both 1000-genome and Genome Aggregation Database (gnomAD).

**Table 3:** List of heterozygous mutations identified.

**Patient**	**Sex**	**Family history**	**Age onset (yrs)**	**Biopsy**	**Nucleotide change**	**Amino acid change**	**Response to therapy**	**Renal Outcome/Time to follow up**	**dbSNP/HGMD ID/ClinVar *In-silico* prediction**
**LAMB2 GENE**									
NS003	F	No	2	MCD^b^	c.3935G/A	p.R1312Q	SRNS	Maintained on ACEI 5 years follow up	rs759882519/not reported/likely pathogenic
NS008	M	No	5	–	c.3152C/T	p.P1051L	SRNS	In remission	rs543606035/not reported/benign
NS094	F	Yes	2	MCD	c.3787G/A	p.E1263L	SRNS	Switched to Tac due to CyA toxicity, partial remission	Not reported/not reported/likely pathogenic
NS118	M	No	3	FSGS^a^	c.2974A/G	p.I992V	SRNS	In remission after CyA	rs529614319/not reported/pathogenic
NS125	F	No	3	FSGS	c.2516T/A	p.L839H	SRNS	ESRD^d^	Not reported/not reported/pathogenic
NS134	F	No	5.5	FSGS	c.1193C/T	p.T398I	SRNS		rs77500937/not reported/likely benign
NS166	F	No	5	FSGS	c.5027G/A	p.G1676E	SRNS	In remission after CyA	Not reported/not reported/pathogenic
NS144	F	No	1.4	–	c.5108G/A	p.R1703H	SRNS		rs771531508/not reported/uncertain significance
NS155		No	3	–	c.4163G/A	p.R1388Q	SRNS		rs146522641/not reported/benign
**NPHS1 GENE**									
NS1401	M	yes	14	FSGS	c.563A/T	p.N188I	SRNS	ESRD	rs145125791/CM020470/likely benign
**NPHS2 GENE**									
NS304	M	no	3	MesPGN^c^	c.708_713delAGAGAG	p.L236LfsX147	SRNS	ESRD	Not reported/not reported/pathogenic

a Focal segmental glomerular sclerosis

b minimal change disease

c mesengial proliferative glomerulonephritis

d *end stage renal disease*.

### Estimation of prevalence and carrier frequencies

We extracted *NPHS1, NPHS2*, and *LAMB2* genes reference and alternate allele counts from two databases; 1000-Genome and Genome Aggregation Database (gnomAD). 1000-Genome browser was used to download the phase 3 data (all population as well as Punjabi from Pakistan; PJL). Data contain 2504 individuals (5008 chromosomes) from 26 populations, whereas, Punjabi population data contain 96 individuals (192 chromosomes) from Lahore, Pakistan. Similarly, all population and South Asian (SAS) allele frequency data was also extracted from the gnomAD database. gnomAD database contains genome/exome sequencing data of 123,136 individuals (all populations) and 15391 exomes data from South-Asian region (SAS, including Pakistanis). We extracted allele frequencies of those variants reported to be disease causing in various studies and are annotated in HGMD and ClinVar databases as known disease causing. *In silico* analyses was performed and we identified 56 known mutations as likely pathogenic. Among these, 18 variants were found in the *NPHS1* gene, 12 in the *NPHS2* gene and 26 variants were noted in the *LAMB2* gene (Table 4). Additionally, we extracted all rare coding region variants that were non-synonymous, non-sense, frameshifting, and were not previously identified as disease causing and scored by using *in-silico* tools. A total of 23 such rare variants (9 in *NPHS1*, 10 in *NPHS2*, and 4 in the *LAMB2* genes) were found in the variant data (Table 5) that were predicted to be deleterious by *In-silico* analysis.

**Table 4:** List of known mutant alleles in the *NPHS1, NPHS2*, and the *LAMB2* genes found in the gnomADe and 1000Genome data.

	**Gene**	**GRCH37 position**	**NCBI dbSNP ID**	**Ref/Alt**	**Consequence**	**gnomADe**	**MAF (%)**	**gnomAD-SAS allele count**	**MAF (%)**	**1K G allele count**	**MAF (%)**	**1KG-PJL allele count**	**MAF (%)**	**SIFT**	**PPH2**	**MA**	**FATHMM**	**CONDEL**	**CONDEL LABEL**
1	NPHS2	1:179520493	rs571452152	A/G	p.Tyr255His	1/245830	0.0004	0/30780	0	1/5008	0.019	–	–	0.01	0.574	2.95	−6.26	0.6718112	D
2	NPHS2	1:179520354	rs768932711	G/A	p.Pro369Leu	2/245844	0.0008	2/30778	0.006		–	–	–	0.15	0.001	0	−6	0.5486173	D
3	NPHS2	1:179520418	rs754243843	T/G	p.Asn348His	1/245938	0.0004	1/30782	0.003		–	–	–	0.04	0.45	1.46	−6.3	0.621615	D
4	NPHS2	1:179520429	rs751105124	A/G	p.Phe344Ser	2/245958	0.0008	2/30780	0.006		–	–	–	0	0.007	1.15	−6.15	0.6076879	D
5	NPHS2	1:179521804	rs770495227	C/A	p.Arg269Ser	1/245354	0.0004	1/30744	0.003		–	–	–	0.21	0.029	0.56	−6.24	0.5838985	D
6	NPHS2	1:179526298	rs771256385	G/T	p.Ser201Tyr	1/246000	0.0004	1/30782	0.003		–	–	–	0.02	0.044	0.8	−6.4	0.6003373	D
7	NPHS2	1:179526308	rs765185151	C/G	p.Glu198Gln	1/245982	0.0004	1/30778	0.003		–	–	–	0.35	0.47	0.75	−3.54	0.5275746	D
8	NPHS2	1:179533863	rs779163992	T/G	p.Ile114Leu	1/246156	0.0004	1/30782	0.003		–	–	–	0.26	0.025	1.21	−6.25	0.6123593	D
9	NPHS2	1:179533901	rs777738678	C/G	p.Cys101Ser	1/246108	0.0004	1/30782	0.003		–	–	–	0	0.131	2.52	−5.92	0.6424912	D
10	NPHS2	1:179526338	rs757665750	C/A	p.Glu188Ter	2/245860	0.0008	2/30780	0.006		–	–	–	–	–	–	–	–	
11	NPHS2	1:179530438	rs778201387	CT/C	p.Arg146GlufsTer35	1/246138	0.0004	1/30782	0.003		–	–	–	–	–	–	–	–	
12	NPHS2	1:179533920	rs759842258	A/AT	p.Ser95IlefsTer8	1/246070	0.0004	1/30770	0.003		–	–	–	–	–	–	–	–	
13	LAMB2	3:49158675	rs769627057	T/C	p.Tyr1794Cys	1/246080	0.0004	1/30780	0.003		–		–	0	0.998	2.69	0.53	0.57388975	D
14	LAMB2	3:49159680	rs779155013	A/G	p.Leu1566Pro	1/245622	0.0004	1/30772	0.003		–		–	0	0.963	2.59	0.92	0.53606262	D
15	LAMB2	3:49161398	rs760892618	C/T	p.Cys1187Tyr	1/244248	0.0004	1/30774	0.003		–		–	0	0.999	4.61	1.39	0.71407755	D
16	LAMB2	3:49161433	rs750381148	C/A	p.Gln1175His	3/244722	0.001	3/30773	0.009		–		–	0.04	0.999	2.08	−0.03	0.52880462	D
17	LAMB2	3:49161479	rs559556131	C/T	p.Gly1160Asp	3/244552	0.001	3/30780	0.009		–		–	0	1	4.67	−1.34	0.85034712	D
18	LAMB2	3:49162278	rs766539657	T/G	p.Ser989Arg	1/246238	0.0004	1/30782	0.003		–		–	0	0.241	2.88	0.06	0.61882555	D
19	LAMB2	3:49162280	rs754732425	C/T	p.Cys988Tyr	3/246234	0.001	2/30782	0.006		–		–	0	0.999	4.6	−3.67	0.77695588	D
20	LAMB2	3:49162299	rs530751136	G/A	p.Arg982Trp	2/246208	0.0008	0/30782	0	1/5008	0.019	1/192	0.52	0.02	0.861	2.31	0.06	0.55138327	D
21	LAMB2	3:49162302	rs778680962	C/T	p.Gly981Ser	1/246222	0.0004	1/30782	0.003		–		–	0.06	1	2.14	0.16	0.5256399	D
22	LAMB2	3:49162334	rs763062098	G/A	p.Pro970Leu	1/246106	0.0004	1/30782	0.003		–		–	0.14	0.999	2.51	−0.05	0.58191315	D
23	LAMB2	3:49162504	rs749808119	G/A	p.Ala940Val	2/243584	0.0008	1/30766	0.003		–		–	0.03	0.999	3.59	−0.14	0.70961854	D
24	LAMB2	3:49162716	rs375392013	C/T	p.Arg897His	2/240634	0.0008	1/30782	0.003		–		–	0.21	0.999	2.16	−0.02	0.53696553	D
25	LAMB2	3:49162762	rs751697643	G/A	p.His882Tyr	1/246028	0.0004	1/30780	0.003		–		–	0.02	0.664	3.89	−0.15	0.74173607	D
26	LAMB2	3:49162811	rs771584138	C/G	p.Gln865His	1/245372	0.0004	1/30780	0.003		–		–	0.12	0.894	2.16	0	0.5357451	D
27	LAMB2	3:49163579	rs764955129	G/A	p.Pro722Leu	1/244568	0.0004	1/30770	0.003		–		–	0.02	0.248	2.69	0.7	0.56367037	D
28	LAMB2	3:49166738	rs751844883	C/T	p.Ser513Asn	2/245150	0.0008	2/30758	0.006		–		–	0.05	0.911	2.26	0.07	0.54547393	D
29	LAMB2	3:49168184	rs747053168	T/C	p.His342Arg	1/246178	0.0004	1/30782	0.003		–		–	0.04	0.63	2.19	0.03	0.53820621	D
30	LAMB2	3:49168388	rs775204900	C/T	p.Gly304Ser	5/246088	0.002	1/30782	0.003		–		–	0.04	1	2.92	−0.2	0.63898086	D
31	LAMB2	3:49168417	rs536441871	G/T	p.Pro294His	1/245872	0.0004	1/30776	0.003	1/5008	0.09	0/192	0	0.05	0.982	3.5	0.06	0.68772865	D
32	LAMB2	3:49168439	rs780152505	C/T	p.Gly287Arg	3/276512	0.001	1/30772	0.003		–		–	0	1	3.78	−0.3	0.738678	D
33	LAMB2	3:49168498	rs772368832	C/T	p.Arg267Gln	7/273782	0.0025	1/30718	0.003		–		–	0.52	0.591	0.98	−0.96	0.52262762	D
34	LAMB2	3:49169033	rs147986864	G/A	p.Arg195Trp	9/275688	0.003	1/30768	0.003		–		–	0	0.979	2.55	−1.13	0.63595261	D
35	LAMB2	3:49169603	rs758877377	G/C	p.Ile135Met	2/246268	0.0008	2/30782	0.006		–		–	0.01	0.961	3.19	−1.3	0.70191895	D
36	LAMB2	3:49169732	rs752886136	C/T	p.Arg119Gln	1/246262	0.0004	1/30782	0.003		–		–	0.59	0.767	3.01	−0.96	0.69118365	D
37	LAMB2	3:49169807	rs767544919	C/T	p.Arg94Gln	3/246272	0.001	2/30782	0.006		–		–	0.18	0.999	2.06	−0.96	0.58205729	D
38	LAMB2	3:49169958	rs776819202	C/T	p.Gly72Asp	1/245660	0.0004	1/30782	0.003		–		–	0.11	0.306	3.06	−0.96	0.69687401	D
39	NPHS1	19:36321826	rs777436326	G/C	p.Pro1172Ala	1/246246	0.0004	0/30782	0	–	–	–	–	0.32	0.563	0.9	−2.97	0.52476152	D
40	NPHS1	19:36330157	rs757169332	G/A	p.Leu1031Phe	1/246246	0.0004	1/30782	0.003	–	–	–	–	0.01	0.34	1.59	−0.85	0.55029744	D
41	NPHS1	19:36330288	rs746380730	T/C	p.Tyr987Cys	1/246264	0.0004	1/30782	0.003	–	–	–	–	0.04	0.982	2.76	0.43	0.58656495	D
42	NPHS1	19:36333170	rs748705495	A/C	p.Val840Gly	1/244238	0.0004	1/30756	0.003	–	–	–	–	0	0.975	1.55	−0.91	0.54873772	D
43	NPHS1	19:36334444	rs750854389	C/A	p.Gly755Val	1/246270	0.0004	1/30782	0.003	–	–	–	–	0.07	0.286	1.94	−1.27	0.54028481	D
44	NPHS1	19:36336347	rs762869410	C/T	p.Gly618Asp	1/245808	0.0004	1/30778	0.003	–	–	–	–	0.04	0.599	2.22	−1.08	0.59841388	D
45	NPHS1	19:36336356	rs751046394	C/A	p.Arg615Leu	2/245746	0.008	2/30782	0.006	–	–	–	–	0.19	0.192	1.25	−0.99	0.53620728	D
46	NPHS1	19:36336408	rs758946523	G/A	p.Pro598Ser	1/234914	0.0004	1/30781	0.003	–	–	–	–	0.64	0.551	1.12	−1.02	0.53008798	D
47	NPHS1	19:36336592	rs764351102	G/A	p.Ser579Tyr	1/245252	0.0004	1/30707	0.003	–	–	–	–	0.09	0.798	2.34	−1.2	0.60973898	D
48	NPHS1	19:36339005	rs749319334	G/A	p.Arg460Trp	6/276520	0.002	1/30766	0.003	–	–	–	–	0.19	0.011	1.74	−1.04	0.54867583	D
49	NPHS1	19:36339010	rs768870360	C/G	p.Gly458Ala	1/245546	0.0004	1/30764	0.003	–	–	–	–	0	0.999	2.11	−2.79	0.56791611	D
50	NPHS1	19:36339250	rs199735886	C/T	p.Arg407Gln	16/246214	0.006	2/30782	0.006	–	–	–	–	0.23	0.271	1.36	−0.96	0.54186854	D
51	NPHS1	19:36339691	rs746481345	G/A	p.Pro340Ser	1/246208	0.0004	1/30782	0.003	–	–	–	–	0.1	1	1.94	−4.43	0.57397387	D
52	NPHS1	19:36339983	rs761786407	C/T	p.Val303Met	1/240840	0.0004	1/30778	0.003	–	–	–	–	0.08	0.571	1	−0.91	0.52460566	D
53	NPHS1	19:36340149	rs752311438	G/T	p.Gln277Lys	1/245308	0.0004	1/30780	0.003	–	–	–	–	1	0.88	1.05	−1.04	0.52596441	D
54	NPHS1	19:36340525	rs779764581	C/A	p.Gln213His	2/246176	0.0008	2/30782	0.006	–	–	–	–	0.05	0.571	1.1	−2.08	0.52665949	D
55	NPHS1	19:36341334	rs779291027	T/C	p.Ile180Met	3/246262	0.001	3/30782	0.009	–	–	–	–	0.15	0.609	1.1	−0.9	0.52979925	D
56	NPHS1	19:36342391	rs761152159	G/C	p.Pro81Arg	1/239854	0.0004	1/30736	0.003	–	–	–	–	0.01	0.996	2.47	0.88	0.52561198	D

D, deleterious

**Table 5:** List of variants alleles in the *NPHS1, NPHS2*, and *LAMB2* genes (scored as likely pathogenic by using *in silico* tools) found in in gnomADe and 1000Genome data.

	**Gene**	**Position**	**dbSNP ID**	**Ref/Alt allele**	**Protein consequence**	**gnomADe**	**MAF (%)**	**gnomAD-SAS allele count**	**MAF (%)**	**1K Allele count**	**MAF (%)**	**1KG-PJL allele count**	**MAF (%)**	**SIFT**	**PPH2**	**MA**	**FATHMM**	**CONDEL**	**CONDEL LABEL**
1	LAMB2	3:49158944	rs760355583	G/A	p.Gln1728Ter	1/246228	0.0004	1/30782	0.003	–	–	–	–	–	–	–	–	–	
2	LAMB2	3:49166461	rs759042337	G/A	p.Arg575Ter	7/241974	0.0028	4/30762	0.013	–	–	–	–	–	–	–	–	–	
3	LAMB2	3:49168473	rs769460144	A/A	p.Tyr275Ter	1/244402	0.0004	1/30750	0.003	–	–	–	–	–	–	–	–	–	
4	LAMB2	3:49167271	rs780041521	C/T	c.1405+1G>A	4/244056	0.0016	1/30746	0.003	–	–	–	–	–	–	–	–	–	
5	NPHS2	1:179520496	rs763818901	G/A	p.Arg322Ter	1/245804	0.0004	1/30782	0.003	–	–	–	–	–	–	–	–	–	–
6	NPHS2	1:179526186	rs748812981	C/A	p.Arg238Ser	4/276486	0.0016	1/30736	0.003	–	–	–	–	0	0.998	3.545	−4.37	0.68850199	D
7	NPHS2	1:179526191	rs146906190	C/G	p.Glu237Gln	205/276570	0.074	4/30748	0.013	1/5008	0.019	0/192	0	0	0.998	3.545	−4.37	0.68850199	D
8	NPHS2	1:179526362	rs74315347	C/T	p.Val180Met	3/245560	0.0012	1/30778	0.003	–	–	–	–	0.02	0.577	1.005	−6.26	0.605115534	D
9	NPHS2	1:179530462	rs74315342	C/T	p.Arg138Gln	159/277072	0.057	2/30782	0.006	–	–	–	–	0.02	0.999	2.28	−6.29	0.641951573	D
10	NPHS2	1:179533825	rs771320565	CT/C	p.Lys126ArgfsTer9	1/246163	0.0004	1/30781	0.003	–	–	–	–	–	–	–	–	–	–
11	NPHS2	1:179520587	rs776016942	C/T	c.874-1G>A	1/245424	0.0004	1/30778	0.003	–	–	–	–	–	–	–	–	–	–
12	NPHS2	1:179520493	rs571452152	A/G	p.Tyr323His	1/245830	0.0004	0/30780	0	1/5008	0.019	1/192	0.5	0.01	0.574	2.945	−6.26	0.671811197	D
13	NPHS2	1:179526301	rs542500942	G/A	p.Ala200Val	5/246006	0.002	4/30780	0.013	1/5008	0.019	1/192	0.5	0.03	0.449	1.47	−3.69	0.554213306	D
14	NPHS2	1:179544873	rs545872093	G/C	p.Pro43Ala	–	–	–		17/5008	0.39	1/192	0.5	0.86	0	−0.205	−5.84	0.529596199	D
15	NPHS1	19:36330221	rs762184939	G/C	p.Tyr1009Ter	2/246268	0.0008	2/30780	0.006	–	–	–	–	–	–	–	–	–	–
16	NPHS1	19:36339690	rs386833861	G/T	p.Pro340His	3/246204	0.0012	3/30782	0.009	–		–	–	0.04	1	1.935	−4.45	0.57449583	D
17	NPHS1	19:36339995	rs753476209	G/A	p.Arg299Cys	2/240156	0.0008	1/30768	0.003	–		–	–	0.02	0.912	1.77	−1	0.549329816	D
18	NPHS1	19:36340176	rs749341977	G/A	p.Arg268Ter	6/275332	0.002	0/30772	0	–		–	–	–	–	–	–	–	–
19	NPHS1	19:36341889	rs386833945	G/A	p.Pro167Leu	1/246044	0.0004	1/30780	0.003	–		–	–	1	1	2.005	−3.06	0.561752557	D
20	NPHS1	19:36339610	rs386833865	G/A	p.Arg367Cys	10/246176	0.004	4/30782	0.013	–	–	–	–	0.01	0.964	1.845	−1.07	0.547062581	D
21	NPHS1	19:36342241	rs386833934	G/A	p.Ala107Val	3/244336	0.0012	2/30718	0.006	–	–	–	–	0.01	0.98	3.22	0.59	0.631325147	D
22	NPHS1	19:36330189	rs749003854	C/A	p.Gly1020Val	6/246268	0.002	6/30782	0.019	–	–	–	–	0	1	3.755	−0.89	0.773421163	D
23	NPHS1	19:36321958	rs267606919	G/A	p.Arg1160Ter	25/246216	0.01	12/30782	0.038	–	–	–	–	–	–	–	–	–	–

An overall NS carrier frequency (CF) of 1:272 (all populations) and 1:278 (SAS population) with a prevalence of 1:295369 (all populations) and 1:308642 (SAS population) was calculated from the known mutant alleles (Table 6) by using the Hardy-Weinberg equation. Inclusion of rare predicted deleterious (but not reported in clinical cohorts; Table 5) alleles to the known mutant alleles list increased the CF to 1:218 (all populations), 1:120 (SAS population) and prevalence to 1:189036 (all populations), and 1:56689 (SAS population; Table 7).

**Table 6:** Carrier frequencies and prevalence based on *NPHS1, NPHS2*, and *LAMB2* known mutations found in the gnomADe and 1000Genome data.

	**gnomAD**	**gnomAD-SAS**	**1KG**	**1KG PJL**	**gnomAD + 1KG**	**gnomAD-SAS + 1KG PJL**
**NPHS2**						
Allele Count	380/256102	15/30772	20in5008	3/192	400/261110	18/30964
Mutant Allele Feq (%)	0.148	0.048	0.399	1.560	0.153	0.058
Prevalence (1 in)	456538	4340278	62814	4109	427186	2972652
Carrier Frq (1 in)	338	1042	126	33	327	863
**LAMB2**						
Allele Count	13/244165	7/30760	0/5008	192	13/249173	7in30952
Mutant Allele Feq (%)	0.005	0.022	0.000	0.000	0.005	0.0220
Prevalence (1 in)	400000000	20661157	–	–	400000000	20661157
Carrier Frq (1 in)	10001	2273	–	–	10001	2273
**NPHS1**						
Allele Count	58/251247	31/30772	0in5008	192	58/256255	31in30964
Mutant Allele Feq (%)	0.023	0.100	0.000	0.000	0.022	0.100
Prevalence (1 in)	18903592	1000000	–	–	20661157	1000000
Carrier Frq (1 in)	2174	501	–	–	2273	501
**NPHS2**+**LAMB2**+**NPHS1**						
Allele Count	451/250505	53/30768	20/5008	3/192	471/255513	56in30960
Mutant Allele Feq (%)	0.18	0.172	0.399	1.56	0.184	0.180
Prevalence (1 in)	308642	338021	62814	4109	295369	308642
Carrier Frq (1 in)	278	291	126	33	272	278

**Table 7:** Carrier frequency and prevalence based on *NPHS1, NPHS2*, and *LAMB2* known plus rare predicted to be pathogenic variants found in the gnomAD and the 1000Genome data.

	**gnomAD**	**gnomAD-SAS**	**1KG**	**1KG-PJL**	**gnomAD + 1KG**	**gnomAD-SAS + 1KG PJL**
**NPHS2**						
Allele Count	395/251020	29/30775	21/5008	3/192	416/256028	32/30967
Mutant Allele Feq. (%)	0.157	0.094	0.419	1.560	0.162	0.103
Prevalence (1 in)	405696	1131734	56960	4109	381039	942596
Carrier Frq. (1 in)	319	532	120	33	309	486
**LAMB2**						
Allele Count	72/246450	40/30768	2/5008	1/192	74/251458	41/30960
Mutant Allele Feq. (%)	0.029	0.130	0.039	0.52	0.029	0.1320
Prevalence (1 in)	11890606	591716	6574622	36982	11890606	573921
Carrier Frq. (1 in)	1725	385	1283	97	1725	379
**NPHS1**						
Allele Count	100/248788	53/30772	0/5008	0/192	100/253796	53/30964
Mutant Allele Feq. (%)	0.040	0.172	0.000	0.000	0.039	0.171
Prevalence (1 in)	6250000	338021	–	–	6574622	341986
Carrier Frq. (1 in)	1251	291	–	–	1283	293
**NPHS2**+**LAMB2**+**NPHS1**						
Allele Count	567/248753	127/30771	23/5008	4/192	590/253761	0.0042
Mutant Allele Feq. (%)	0.227	0.412	0.459	2.08	0.23	0.420
Prevalence (1 in)	194065	58912	47465	2311	189036	56689
Carrier Frq. (1 in)	221	122	109	25	218	120

## Discussion

SRNS cases are challenging in the manner of highly variable clinical outcomes where 50% of the children develop ESRD within 15 years of life (Mekahli et al., 2009; Zagury et al., 2013). It constitutes the second most frequent cause of ESRD in the first two decades of life (North-American Pediatric Renal Trials and Collaborative Studies, NAPRTCS, 2008). In a previous analysis we have shown that mutations in the *NPHS1* and the *NPHS2* were not very common among CNS, SRNS, and familial cases in our population (Abid et al., 2012). Our results showed a low prevalence of disease causing mutations in the *NPHS1* (22% early onset, 5.5% overall) and *NPHS2* (3.3% early onset and 3.4% overall) genes in the studied NS population from Pakistan as compared to the other populations (Sadowski et al., 2015). To further extend the spectrum of the disease causing mutations in other NS causing genes, we selected a cohort of patients in whom the *NPHS1* and *NPHS2* genes have been excluded. We identified 50 such cases from our previous cohort including 14 new cases recruited for the *NPHS1* and *NPHS2* gene screening. Hinkes et al. (2007) have shown that 85% of the SRNS cases with congenital onset and 66% with infantile onset can be explained by mutations in the *NPHS1, NPHS2, WT1*, and *LAMB2* genes. Matejas et al. (2010) indicated that the analysis of *LAMB2* gene which is mutated in Pierson syndrome could be included in the diagnostics of early onset NS in the absence of extra renal abnormalities. Based on the screening algorithm presented by Hinkes et al. (2007) and Matejas et al. (2010) we selected *LAMB2* and *WT1* genes along with the *NPHS1* and the *NPHS2* genes for screening in our cohort.

Mutations identified in the *LAMB2* gene (Table 2) in our cohort indicated that the *LAMB2* gene should be included in the analysis algorithm as previously opposed by Santín et al. (2011) who excluded this gene from the algorithm introduced for molecular-genetic diagnostics of non-syndromic childhood onset primary SRNS. They also proposed the analysis of exons 8 and 9 of the *WT1* gene in this algorithm; however upon screening of the whole *WT1* gene, we did not find a single mutation in our isolated NS cases. Lipska et al. (2014) reported *WT1* missense mutations associated with diffuse mesangial sclerosis (74%), early-onset SRNS and rapid progression to ESRD. In a cohort of Saudi Arabian families with childhood NS, disease causing mutations in the *NPHS1* gene were identified in 12% (6/49) cases and in *NPHS2* mutations were found in 22%(11/49) cases (Al-Hamed et al., 2013). Similarly, screening of 36 SRNS patients from the UK renal registry, molecular analysis revealed causative *NPHS1* and *NPHS2* gene mutations in 14% (5/36) and 8% (3/36) of the patients (McCarthy et al., 2013). One interesting observation of these two studies was the absence of *LAMB2* and *WT1* gene mutation in the two later studies.

Several recent studies have also reported gene panel screening with all the known genes in large cohorts using the next generation sequencing (NGS) technology (Bullich et al., 2015; Sadowski et al., 2015). These reports present a single gene cause of the disease in 29–36% of the patients. In comparison to these International cohort results, Wang et al. (2017) have identified a single gene cause of NS in 28.3% cases in Chinese cohort. The commonly mutated genes were *ADCK4* (6.67%), *NPHS1* (5.83%), *WT1* (5.83%), and *NPHS2* (3.33%) among Chinese populations. The differences between the Chinese study (Wang et al., 2017) and the Sadowski et al. (2015) study are significant, where the most common disease causing gene in Chinese cohort was *ADCK4*. No *NPHS2* gene mutation was detected in the CNS or infantile NS cases in the Chinese cohort consistent with the results of Japanese, Korean, African-American SRNS populations and Pakistanis (Chernin et al., 2008; Cho et al., 2008; Wang et al., 2017). This was in contrary to some other reports, where mutations in the *NPHS2* gene was found in 12% in Spanish population (Santín et al., 2011), 5.7–12.7% in an International cohort (Bullich et al., 2015; Sadowski et al., 2015), 37.5% in a European cohort of CNS and infantile onset NS (Hinkes et al., 2007).

The data indicate marked ethnic and geographical differences in the etiology of disease. The exact cause of this wide variation in the prevalence of genetic abnormalities in different studies is not clear, but may reflect ethnic differences, the use of different inclusion criteria, the differences in the methodologies used for the detection of mutations in these genes, and the number of genes screened for mutations. In the current study, we included only those cases of pediatric NS known to have high prevalence of genetic abnormalities. The observed low prevalence of nephrin, podocin, laminin B2 and Wilm's tumor-1gene mutations in CNS, infantile, and childhood SRNS is in marked contrast to the findings from the studies from Europe, and US, where the prevalence of these gene mutations are high (Hinkes et al., 2007; Matejas et al., 2010). However, our findings are concordant with the findings from a few regional studies, such as studies from China, Korea, Japan, and African American children (Sako et al., 2005; Chernin et al., 2008; Cho et al., 2008; Wang et al., 2017). The later study did not find any mutations in *NPHS2* and exons 8 and 9 of the *WT1* genes in African American cohort.

We did not find any significant correlation of the mutations in either gene with the age of the patients, clinical subtypes, gender, histology of the lesions, prognosis, or family history. Similar findings have been reported by most other investigators as well. This most probably is due to the small number of patients with each distinct type of mutations. Large studies with longer follow up may be helpful in this context.

We calculated the NS prevalence based on frequencies of genes in large population databases; the 1000 Genome and gnomAD. We have found an overall carrier frequency of 1 in 218 worldwide and 1 in 120 in South-Asian populations. Worldwide prevalence was found to be 1 in 189,036 and 1 in 56,689 in South Asian populations (Table 7). According to the literature, idiopathic nephrotic syndrome has a reported incidence of two to seven cases per 100,000 children and a prevalence of 16 cases per 100,000 (Eddy and Symons, 2003). There is also an epidemiological evidence of a higher incidence of nephrotic syndrome in children from South Asia (McKinney et al., 2001). The current estimates of prevalence of NS in three genes population data showed a much higher prevalence in SAS populations than the clinical studies estimations; however the worldwide data showed a much lesser prevalence according to current population data. There is no proper registry maintained for the prevalence of NS in Pakistani children. However, some individual single center studies presented local prevalence (Ali et al., 2011; Mubarak et al., 2012; Shah et al., 2015; Imtiaz et al., 2016). According to these estimates, NS is the most prevalent clinical presentation represented 50–74% of all the children presented with renal diseases (Ali et al., 2011; Imtiaz et al., 2016). The most common histo-pathological presentation was minimal change disease (MCD; 24.09–30.3%) followed by FSGS (18.3–38%) in these subjects (Ali et al., 2011; Mubarak et al., 2012; Imtiaz et al., 2016).

There are a few limitations of this study. A small sized patient cohort screening may reduce the chances of identification of disease mutations. However our cohort is a heterogeneous cohort with patients collected from all major ethnicities in Pakistan. SIUT is a tertiary care center, where patients are referred from all over Pakistan representing all the major ethnicities in the country. We only screened four genes reported to cause NS in children as we hypothesized this study according to the observations of some previously conducted studies (Hinkes et al., 2007; Zenker et al., 2009; Matejas et al., 2010; Santín et al., 2011). There are now more than 40 genes reported to cause different types of NS (Lipska et al., 2014; Bullich et al., 2015; Sadowski et al., 2015). It is therefore, necessary to design a comprehensive study to screen all the known disease causing gene for a better understanding of the disease etiology in this cohort.

In conclusion, we have observed a low prevalence of disease causing mutations in three major disease causing genes in our NS population and no mutations in the *WT1* gene in early onset NS cases. The data indicated that genetic screening strategies put forward for several international populations may not be appropriate for this cohort. Therefore, a comprehensive screening for all the known genes is required which will help in improved management of the disease and will provide a reference population for gene screening within evolutionary related populations of this region.

## Ethics statement

This study was carried out in accordance with the recommendations of Centre for Biomedical Ethics and Culture. The protocol was approved by the Centre for Biomedical Ethics and Research. Written informed consent was obtained from the parents of the subjects in accordance with the Declaration of Helsinki.

## Author contributions

AA and SK conceived and designed the study. AA, SS, and MS performed the study. AA analyzed and prepared the manuscript. AL and SH provided sample and clinical data.

### Conflict of interest statement

The authors declare that the research was conducted in the absence of any commercial or financial relationships that could be construed as a potential conflict of interest.
